# Supplementary material for: Estimating allele frequencies, ancestry proportions and genotype likelihoods in the presence of mapping bias
Source: G3 (Bethesda). 2025 Jul 30;15(10):jkaf172. doi: 10.1093/g3journal/jkaf172 (PMC12506655; doi:10.1093/g3journal/jkaf172)
Supplement: jkaf172_Supplementary_Data [file jkaf172_supplementary_data.zip › Supplemental_Figures_and_Tables_G3-2025-406068.pdf]

Estimating allele frequencies, ancestry proportions and genotype likelihoods in the presence of mapping bias

Supplementary Material

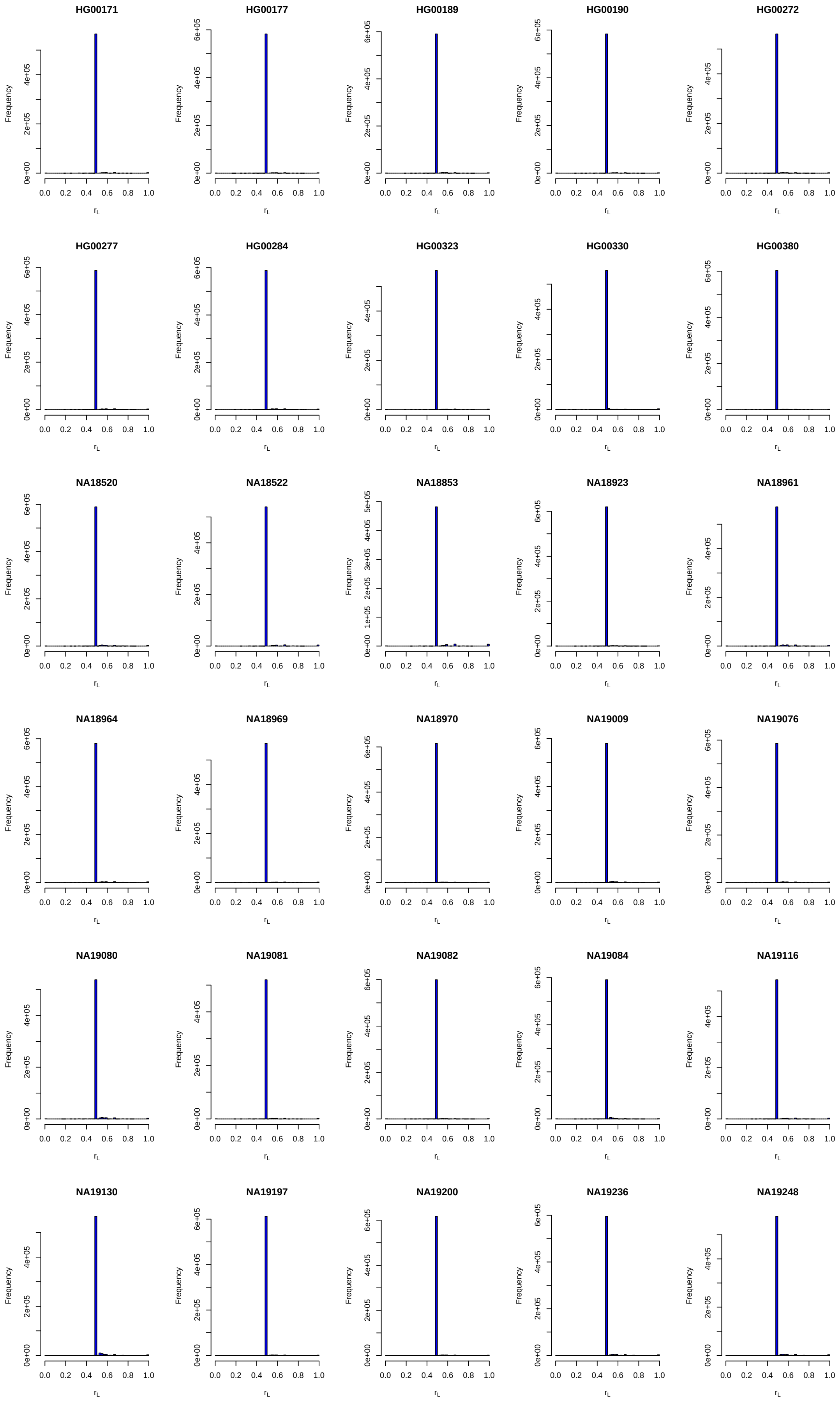

Supplementary Figure 1: Histograms of r_L for all 30 KGP individuals.

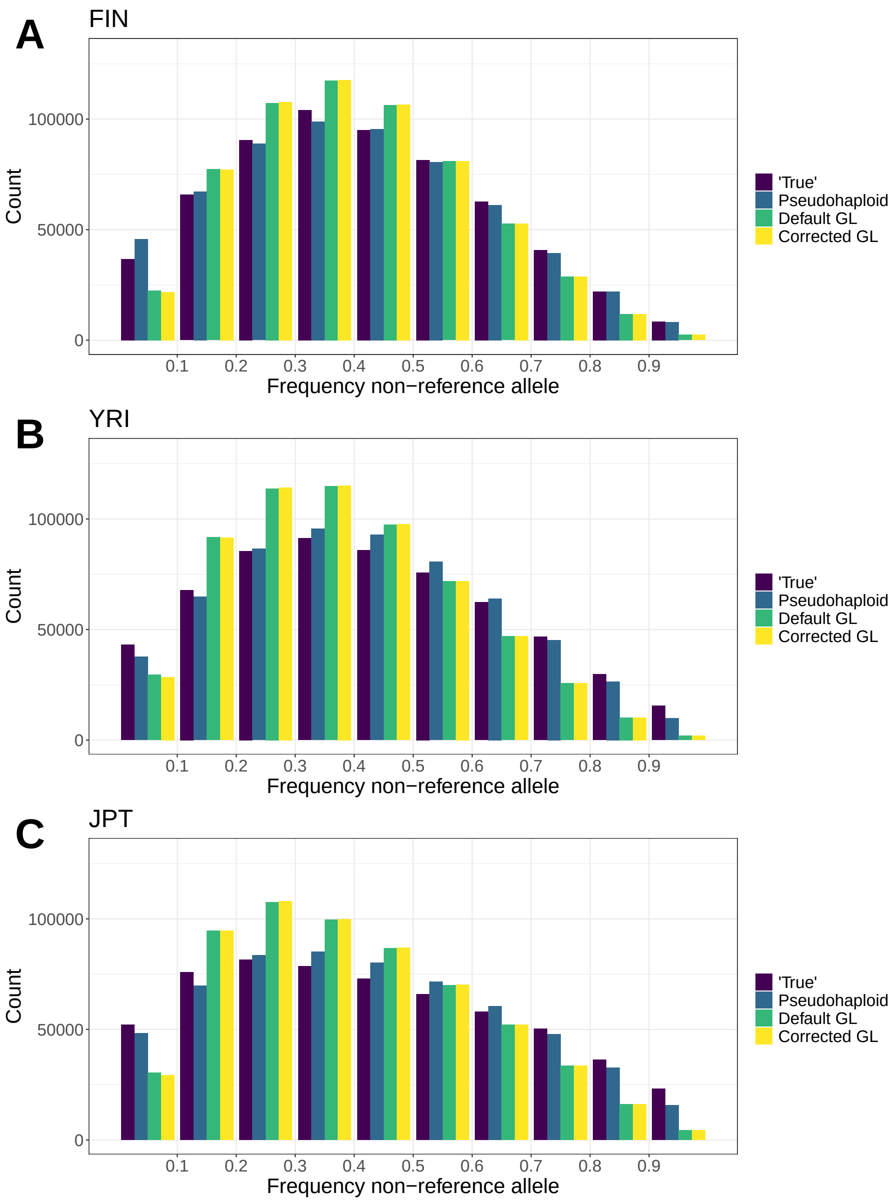

Supplementary Figure 2: Binned spectrum of non-reference alleles in FIN (A), YRI (B) and JPT (C) for the four different estimation methods. Note that the specific ascertainment of common SNPs in the joint genotyping data contributes to the enrichment of variants with (true) intermediate frequencies.

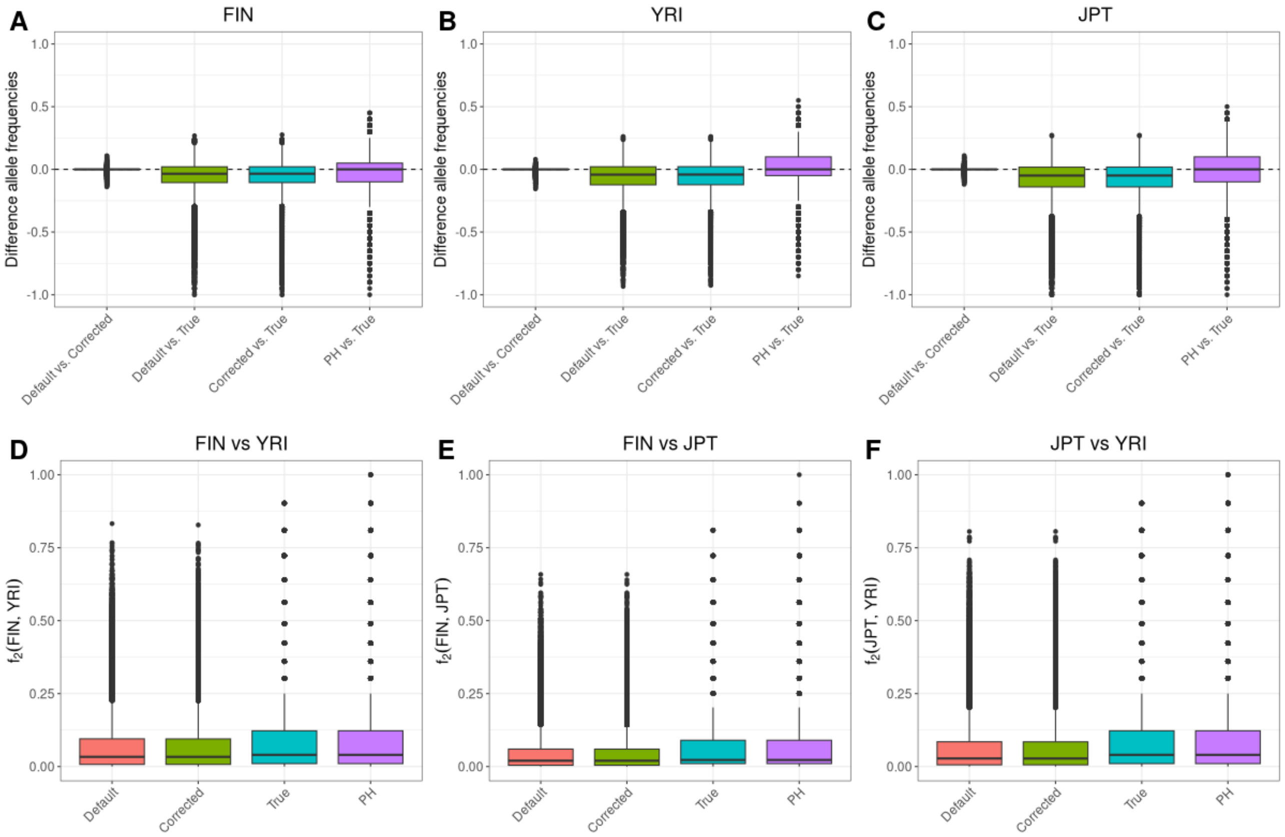

Supplementary Figure 3: Differences in allele frequency estimates in the parts of the reference genome attributed to African ancestry. Boxplots for the differences between default genotype likelihood-based estimates and corrected genotype likelihood-based estimates, default genotype likelihood-based estimates and SNP array-based estimates, corrected genotype likelihood-based estimates, pseudohaploid (PH) genotype-based and SNP array-based estimates (A) in the FIN population and (B) in the YRI population. (C) is showing boxplots of the per-site population differentiation (measured as f_2 statistic) for the four allele frequency estimates.

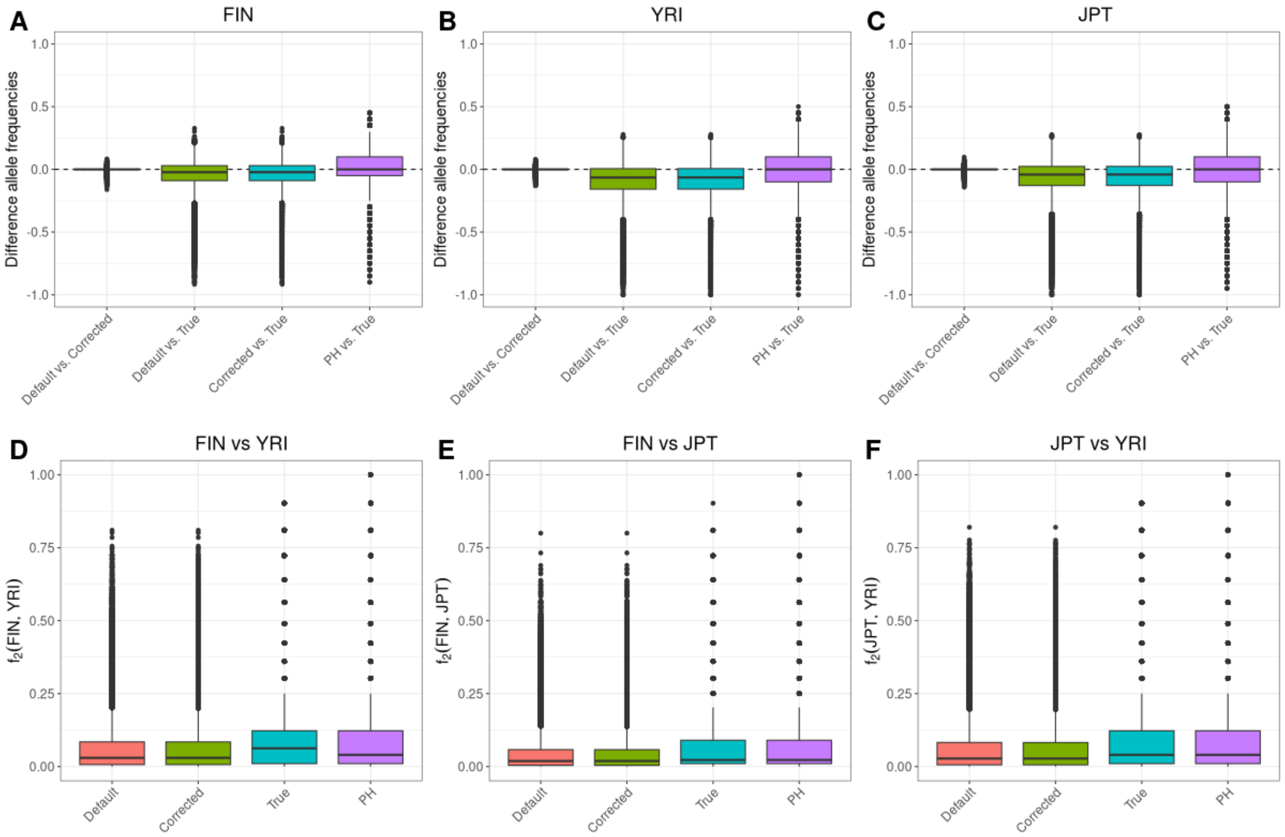

Supplementary Figure 4: Differences in allele frequency estimates in the parts of the reference genome attributed to European ancestry. Boxplots for the differences between default genotype likelihood-based estimates and corrected genotype likelihood-based estimates, default genotype likelihood-based estimates and SNP array-based estimates, corrected genotype likelihood-based estimates, pseudohaploid (PH) genotype-based and SNP array-based estimates (A) in the FIN population and (B) in the YRI population. (C) is showing boxplots of the per-site population differentiation (measured as f_2 statistic) for the four allele frequency estimates.

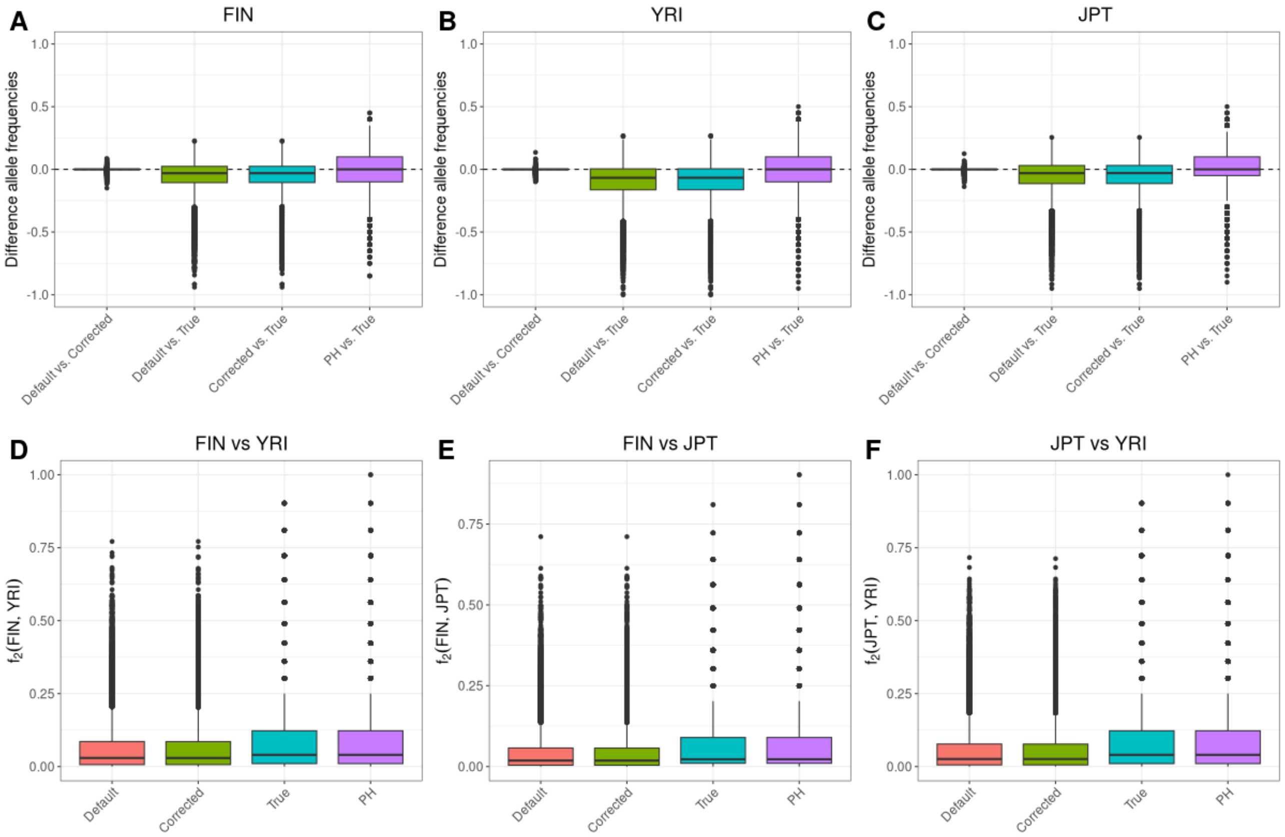

Supplementary Figure 5: Differences in allele frequency estimates in the parts of the reference genome attributed to East Asian ancestry. Boxplots for the differences between default genotype likelihood-based estimates and corrected genotype likelihood-based estimates, default genotype likelihood-based estimates and SNP array-based estimates, corrected genotype likelihood-based estimates, pseudohaploid (PH) genotype-based and SNP array-based estimates (A) in the FIN population and (B) in the YRI population. (C) is showing boxplots of the per-site population differentiation (measured as f_2 statistic) for the four allele frequency estimates.

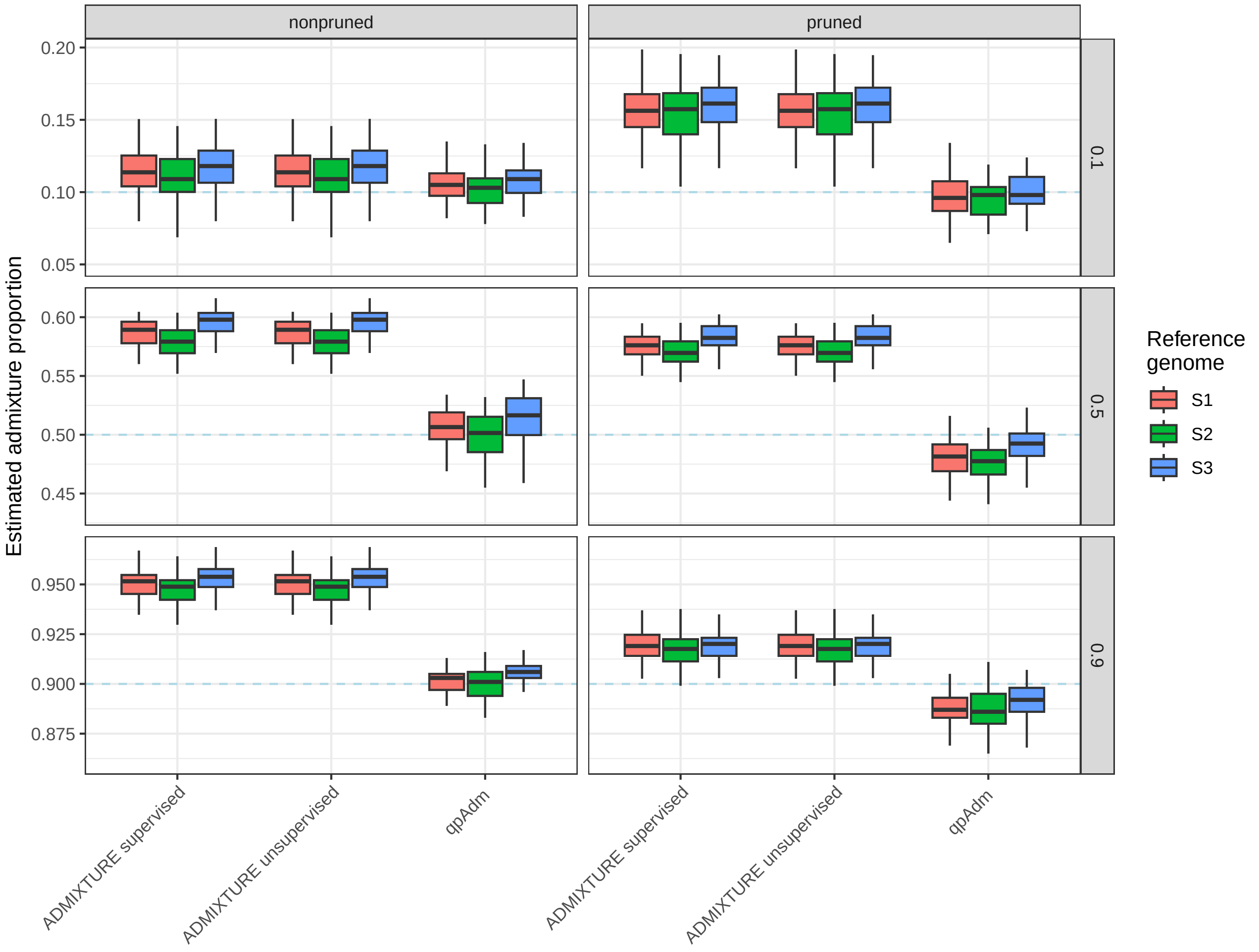

Supplementary Figure 6: Simulation results for genotype call based methods using $t_{123} = 20000$ generations and a sequencing depth of 0.5X. Dashed blue lines represent the simulated admixture proportions, i.e. the gene flow received from $S3$ 500 generations ago.

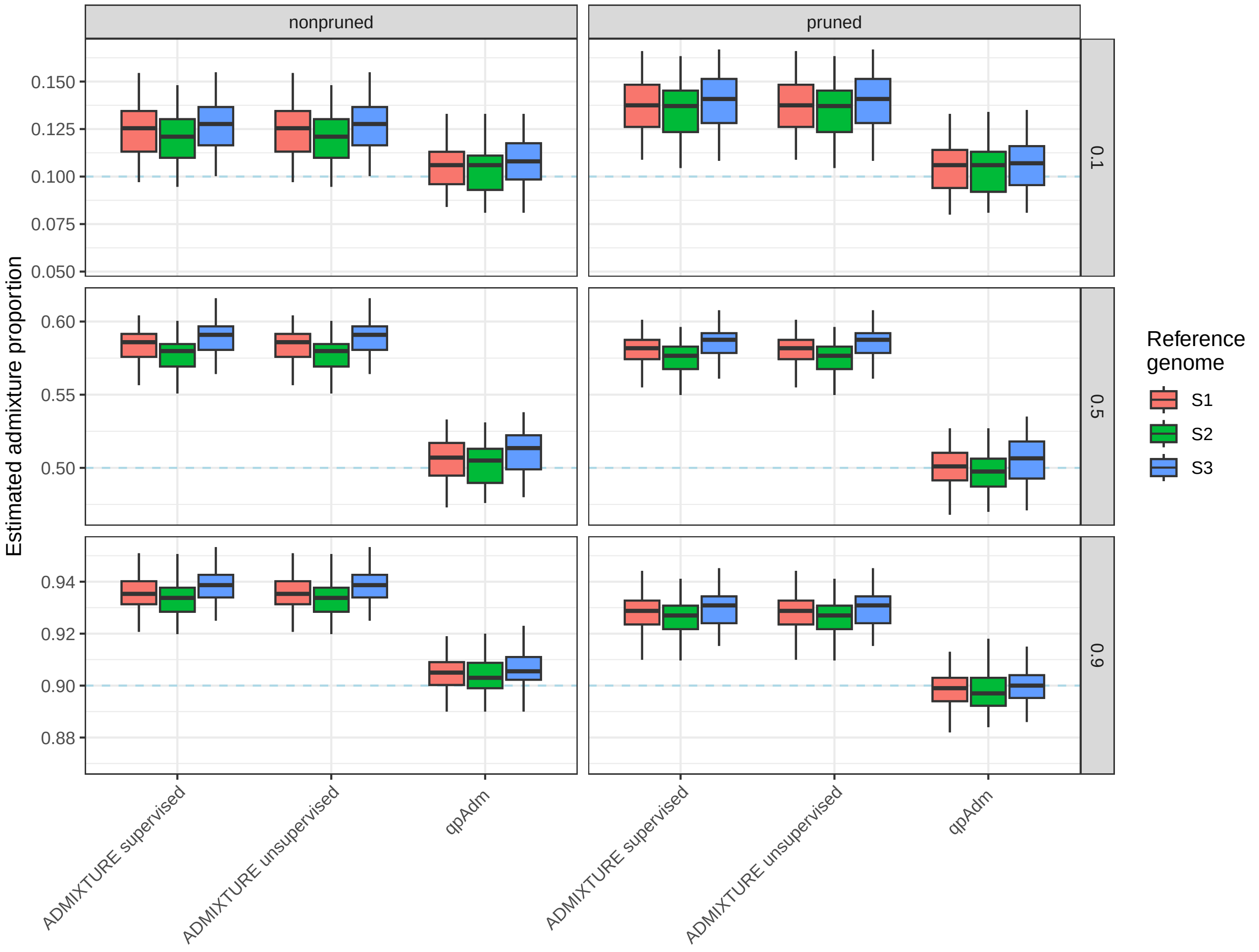

Supplementary Figure 7: Simulation results for genotype call based methods using $t_{123} = 20000$ generations and a sequencing depth of 2.0X. Dashed blue lines represent the simulated admixture proportions, i.e. the gene flow received from $S3$ 500 generations ago.

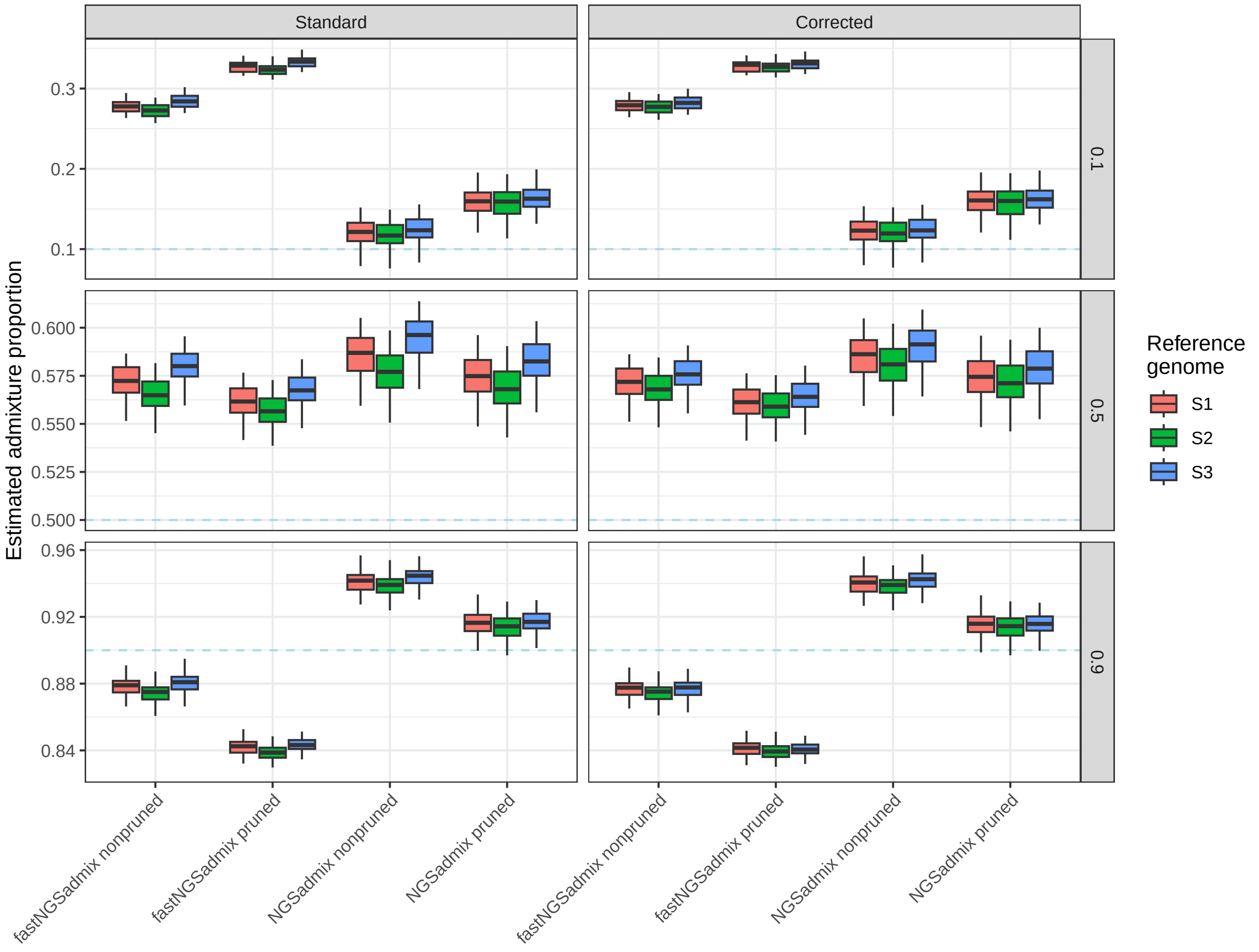

Supplementary Figure 8: Simulation results for genotype likelihood based methods using $t_{123} = 20000$ generations and a sequencing depth of 0.5X. Dashed blue lines represent the simulated admixture proportions, i.e. the gene flow received from $S3$ 500 generations ago.

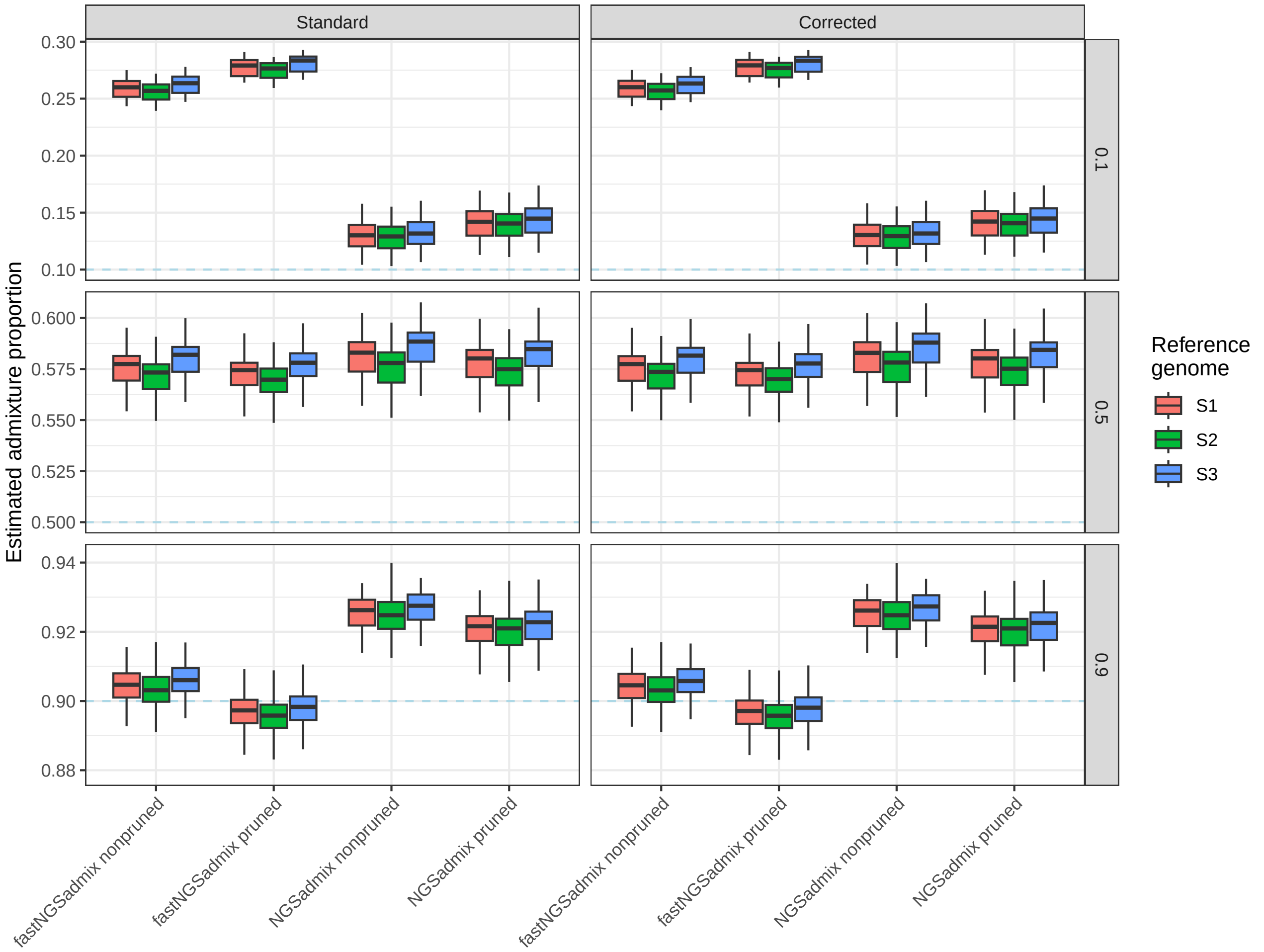

Supplementary Figure 9: Simulation results for genotype likelihood based methods using $t_{123} = 20000$ generations and a sequencing depth of 2.0X. Dashed blue lines represent the simulated admixture proportions, i.e. the gene flow received from $S3$ 500 generations ago.

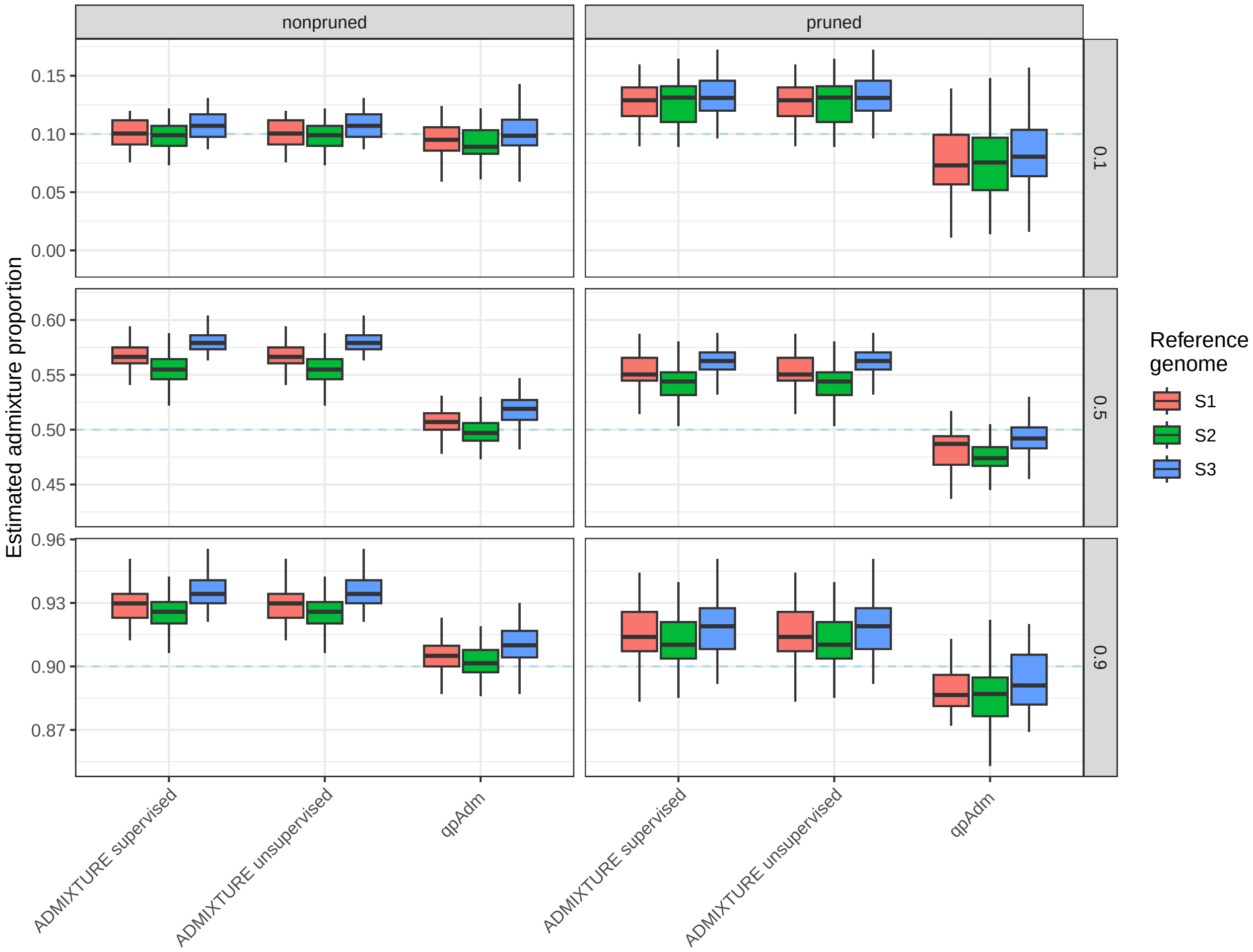

Supplementary Figure 10: Simulation results for genotype call based methods using $t_{123} = 50000$ generations and a sequencing depth of 2.0X. Dashed blue lines represent the simulated admixture proportions, i.e. the gene flow received from $S3$ 500 generations ago.

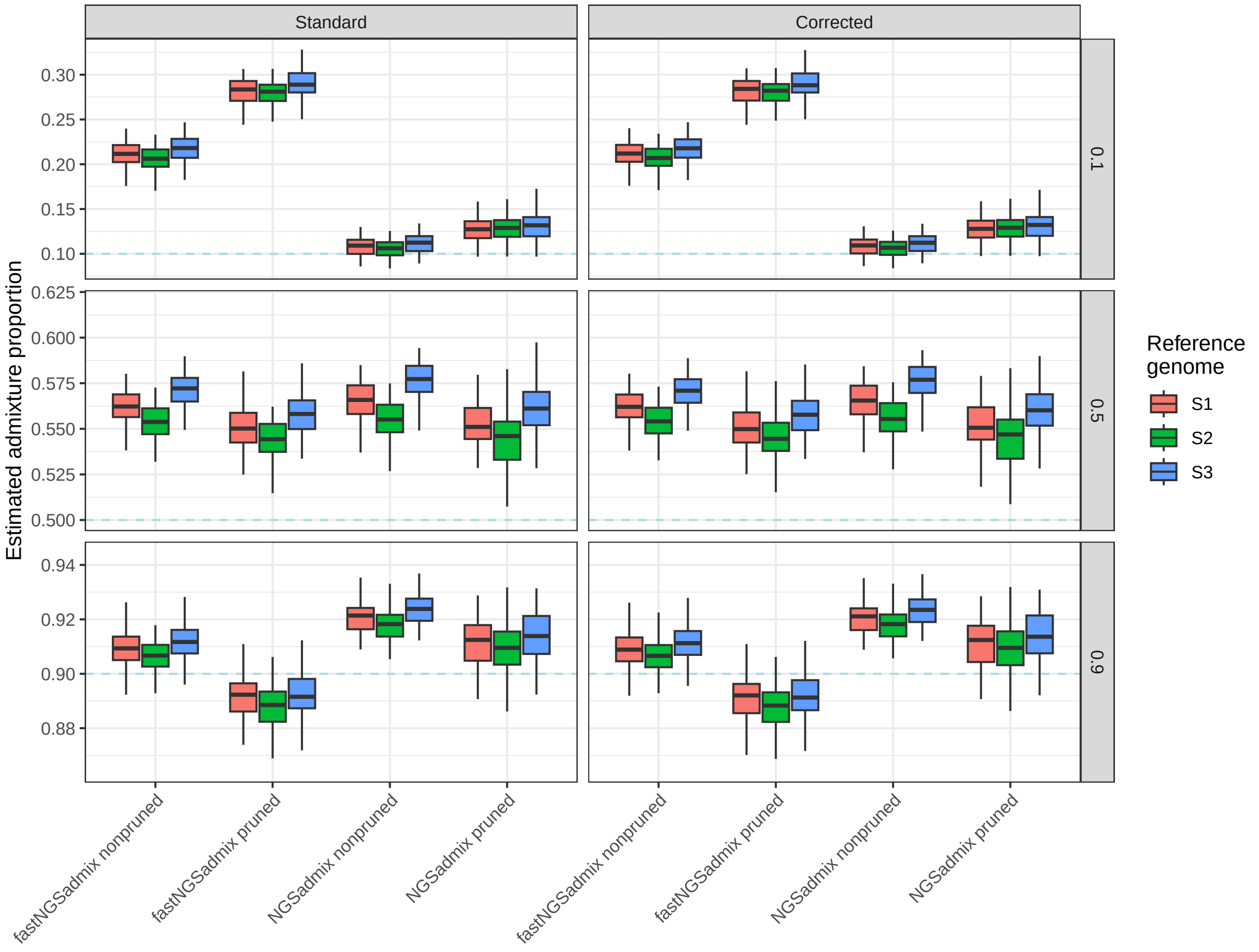

Supplementary Figure 11: Simulation results for genotype likelihood based methods using $t_{123} = 50000$ generations and a sequencing depth of 2.0X. Dashed blue lines represent the simulated admixture proportions, i.e. the gene flow received from $S3$ 500 generations ago.

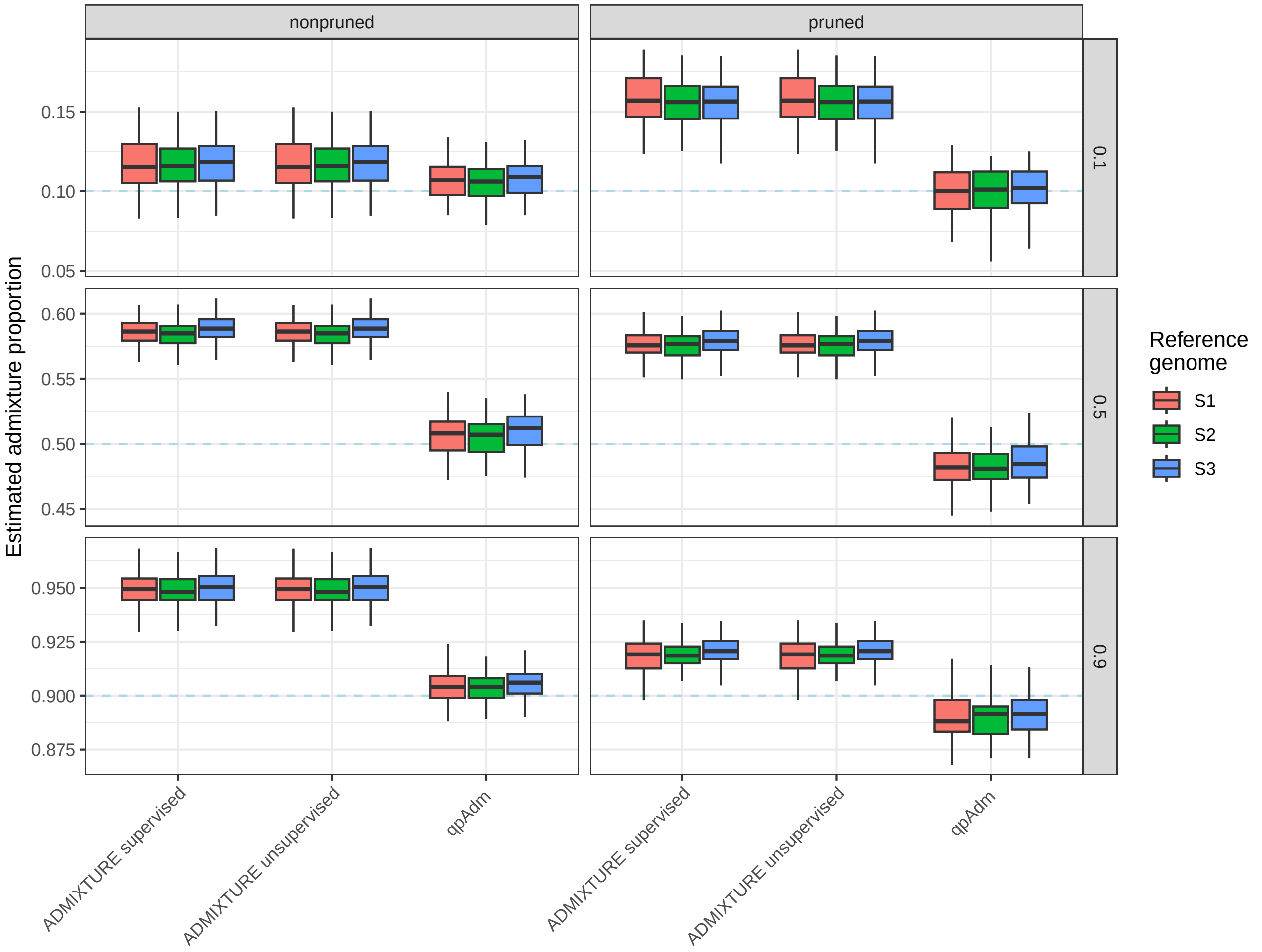

Supplementary Figure 12: Simulation results for genotype call based methods using $t_{123} = 20000$ generations and a sequencing depth of 0.5X. Dashed blue lines represent the simulated admixture proportions, i.e. the gene flow received from $S3$ 500 generations ago. For this run, the mapping quality threshold was set to 25 instead of 30 as in all other runs.

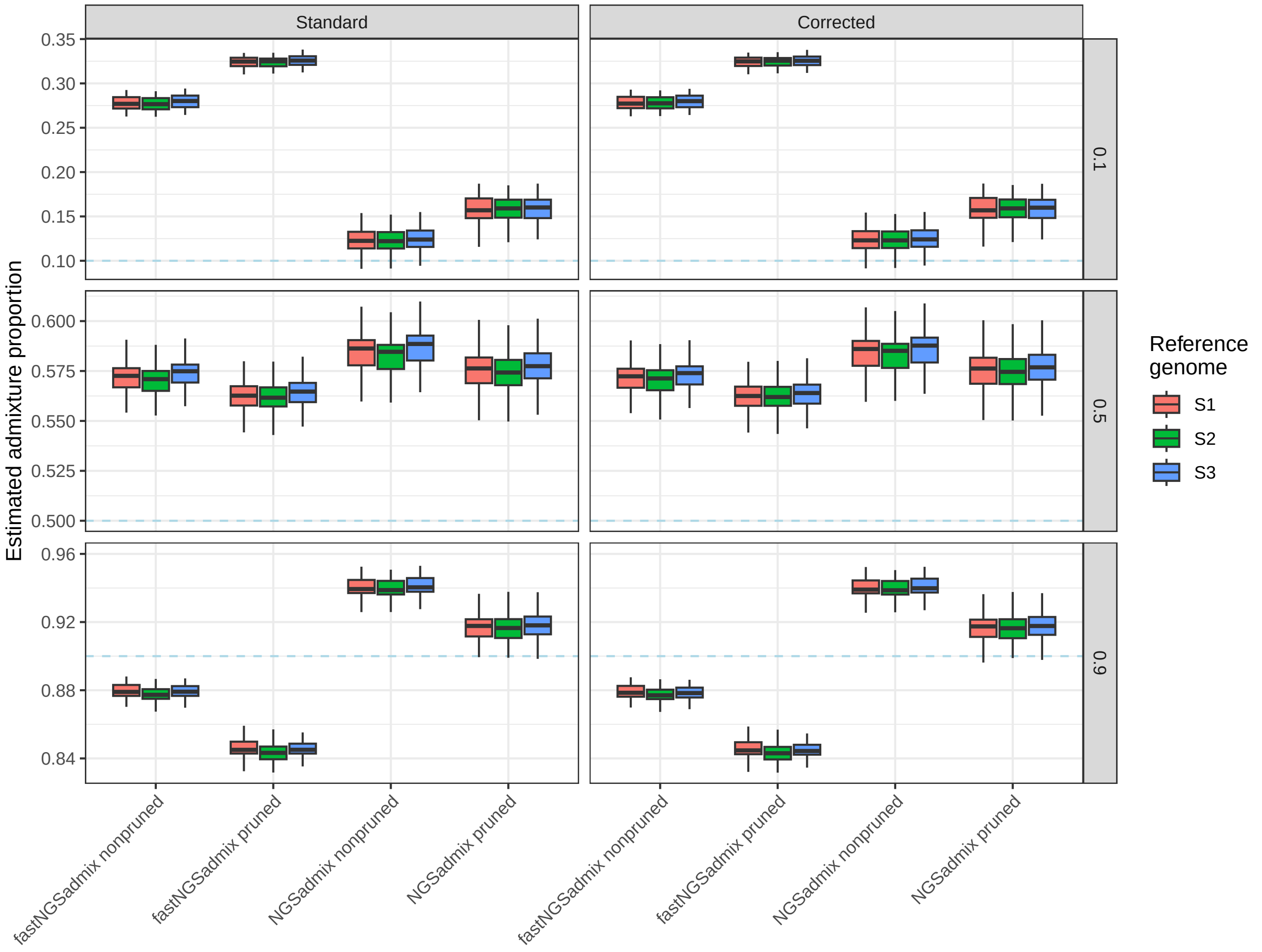

Supplementary Figure 13: Simulation results for genotype likelihood based methods using $t_{123} = 20000$ generations and a sequencing depth of 0.5X. Dashed blue lines represent the simulated admixture proportions, i.e. the gene flow received from $S3$ 500 generations ago. For this run, the mapping quality threshold was set to 25 instead of 30 as in all other runs.

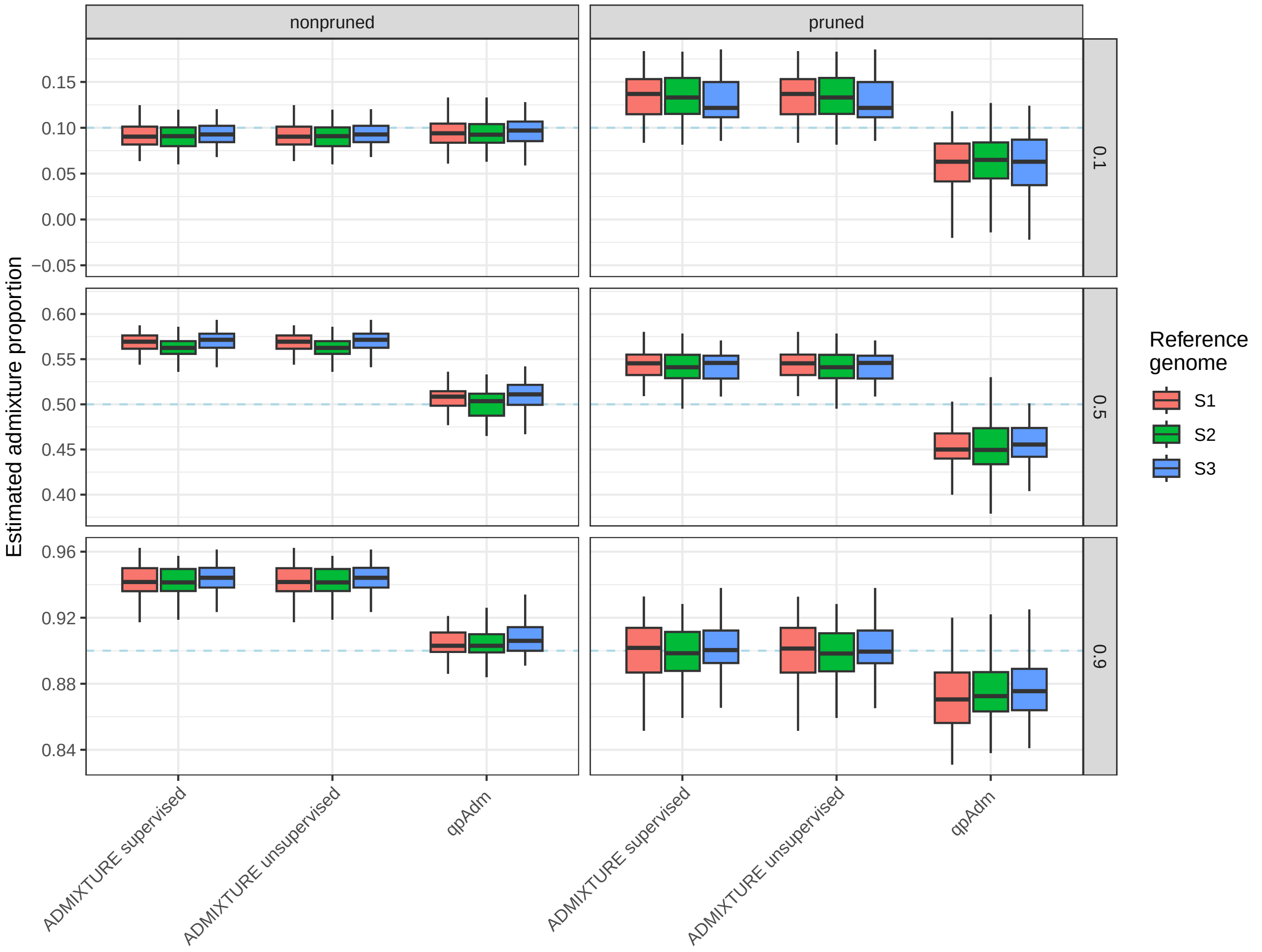

Supplementary Figure 14: Simulation results for genotype call based methods using $t_{123} = 50000$ generations and a sequencing depth of 0.5X. Dashed blue lines represent the simulated admixture proportions, i.e. the gene flow received from $S3$ 500 generations ago. For this run, the mapping quality threshold was set to 25 instead of 30 as in all other runs.

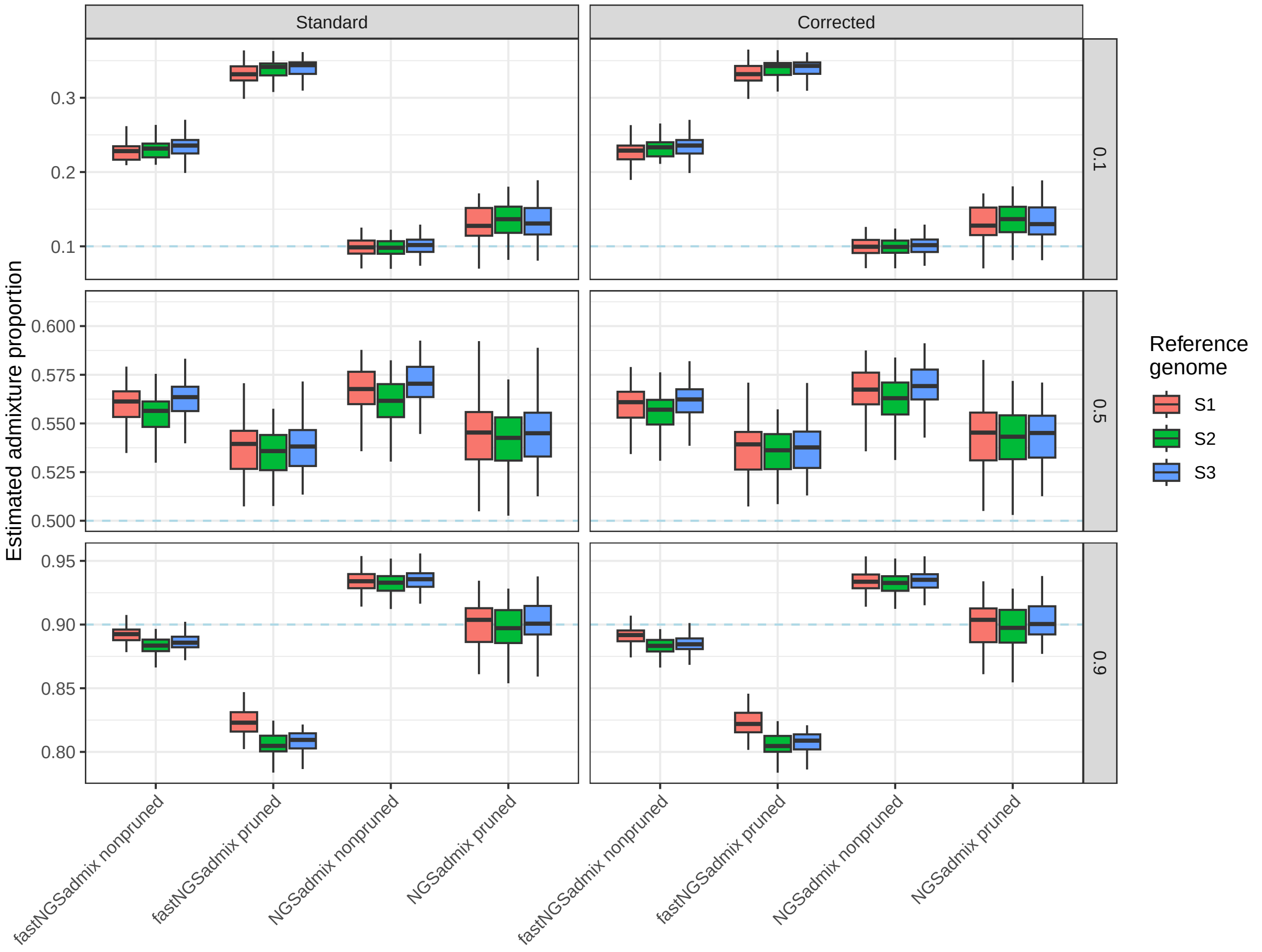

Supplementary Figure 15: Simulation results for genotype likelihood based methods using $t_{123} = 50000$ generations and a sequencing depth of 0.5X. Dashed blue lines represent the simulated admixture proportions, i.e. the gene flow received from $S3$ 500 generations ago. For this run, the mapping quality threshold was set to 25 instead of 30 as in all other runs.

Supplementary Table 1: Command line parameters used for ANGSD call to produce pseudohaploid genotype calls.

ANGSD command line flag	Setting	Consequence
-checkBamHeaders	0	deactivate checking the headers of the BAM files
-doHaploCall	1	sample a single base only
-doCounts	1	needed to determine the most common base
-doGeno	-4	do not print genotypes
-doPost	2	estimate the posterior genotype probability assuming a uniform prior, output files not used
-doPlink	2	produce output in tfam/tped format
-minMapQ	30	set the minimum mapping quality to 30
-minQ	30	set the minimum base quality
-doMajorMinor	1	to infer major and minor allele from genotype likelihoods
-GL	2	calculate GATK genotype likelihood, output files not used
-domaf	1	calculate allele frequencies with fixed major and minor alleles

Supplementary Table 2: 1000 genomes individuals used for the analysis of empirical data.

Individual	Population	Autosomal sequencing depth	Average original read length	Average r_L
HG00171	FIN	3.12803	108	0.5031
HG00177	FIN	3.43327	108	0.5023
HG00189	FIN	3.48314	108	0.5026
HG00190	FIN	3.089	108	0.5023
HG00272	FIN	3.61242	108	0.5027
HG00277	FIN	3.86275	76	0.5052
HG00284	FIN	4.08807	76	0.5052
HG00323	FIN	2.80008	89.19	0.5035
HG00330	FIN	13.9648	90.22	0.5045
HG00380	FIN	3.45273	100	0.502
NA18961	JPT	3.48611	76	0.5067
NA18964	JPT	3.333	76	0.5052
NA18969	JPT	2.6653	100	0.5026
NA18970	JPT	4.47082	100	0.502
NA19009	JPT	3.94626	108	0.5033
NA19076	JPT	3.50604	108	0.5029
NA19080	JPT	3.84401	108	0.5055
NA19081	JPT	2.60827	108	0.5034
NA19082	JPT	3.58866	108	0.5018
NA19084	JPT	4.37475	108	0.5026
NA18520	YRI	3.99207	76	0.5057
NA18522	YRI	2.55368	76	0.5066
NA18853	YRI	2.56291	76	0.5099
NA18923	YRI	4.42742	100	0.5019
NA19116	YRI	3.03829	82.51	0.5056
NA19130	YRI	4.97799	76	0.5061
NA19197	YRI	4.19443	100	0.5021
NA19200	YRI	4.22902	100	0.502
NA19236	YRI	4.21535	76	0.5055
NA19248	YRI	4.24979	76	0.5058

Supplementary Table 3: Average read balances for the 1000 genomes populations used for the analysis of empirical data.

Population	Average r_L
FIN	0.50334
JPT	0.5036
YRI	0.50512
